# Serum TIMP-1 concentrations are associated with cancer incidence and cancer-related mortality in a population-based cohort

**DOI:** 10.1038/s41598-026-52354-5

**Published:** 2026-05-20

**Authors:** M. T. Nieminen, E. Brandt, A. S. Havulinna, O. Kambur, T. Tervahartiala, T. Zeller, S. Blankenberg, V. Salomaa, T. Sorsa, P. J. Pussinen, A. Salminen

**Affiliations:** 1 Oral and Maxillofacial Diseases, University of Helsinki and Helsinki University Hospital, Helsinki, Finland; 2 Department of Otorhinolaryngology, Head and Neck Surgery, University of Helsinki and Helsinki University Hospital, Helsinki, Finland; 3 Institute for Molecular Medicine Finland, FIMM-HiLIFE, Helsinki, Finland; 4 Department of Computing, University of Turku, Turku, Finland; 5 School of Medicine, Institute of Dentistry, University of Eastern Finland, Kuopio, Finland; 6 Clinic for General and Interventional Cardiology, University Heart Center Hamburg, Hamburg, Germany; 7 German Center for Cardiovascular Research (DZHK E.V), Partner Site Hamburg/Lübeck/Kiel, Germany; 8 Department of Medicine, University of Turku, Turku, Finland; 9 Department of Dental Medicine, Karolinska Institutet, Huddinge, Sweden

**Keywords:** Matrix metalloproteinase 8, Tissue inhibitor of matrix metalloproteinase 1, Cancer, Biomarker, Tumor, Biomarkers, Cancer, Diseases, Oncology, Risk factors

## Abstract

The tumor microenvironment is regulated by matrix metalloproteinases (MMPs) and their tissue inhibitors (TIMPs); however, their utility as cancer biomarkers remains unclear. We investigated the associations of serum MMP-8, TIMP-1, and the MMP-8/TIMP-1 ratio with prevalent and incident cancer in 8448 individuals aged 25–74 years from the FINRISK97 cohort. Participants were followed for incident cancer (n = 1618) for 26 years through the Finnish Cancer Registry. Serum MMP-8 and TIMP-1 concentrations were measured at baseline using immunoassays. Serum TIMP-1 levels were significantly higher in individuals with prevalent and incident cancer, including colorectal and lung cancer, whereas lower MMP-8 levels were observed in colorectal and prostate cancer. Accordingly, the MMP-8/TIMP-1 ratio was reduced in individuals with incident cancer. In age-stratified models adjusted for sex, smoking, and BMI, elevated TIMP-1 levels were strongly associated with incident cancer in participants aged 55–64 years (HR, 95% CI 6.36, 2.50–16.16) and ≥ 65 years (6.23, 1.67–23.21), and with cancer mortality (29.7, 8.25–106 and 18.4, 3.64–93.6). MMP-8 and the MMP-8/TIMP-1 ratio showed weaker associations with incident cancer and cancer mortality only in the 45–54-year age group. These findings support TIMP-1 as a potential biomarker for cancer risk and mortality.

**Supplementary Information:**

The online version contains supplementary material available at 10.1038/s41598-026-52354-5.

## Introduction

Cancer continues to be a significant global health challenge, highlighting the urgent demand for biomarkers that can facilitate early detection of the disease. It is estimated that approximately one in five men or women develop cancer during the lifetime^1^. Cancer biomarkers have potential to help with early diagnostics, reducing the scope and cost of cancer care, and improving life expectancy and reducing cancer mortality^2^.

Matrix metalloproteinases (MMPs) comprise a family of over twenty different endopeptidases capable of degrading nearly all components of the extracellular matrix (ECM), playing crucial roles in numerous physiological and pathological processes^3,4^. A fundamental characteristic of malignant tumors is their ability to infiltrate surrounding tissues and metastasize. Consequently, the breakdown and remodeling of the ECM are essential for carcinogenesis, with MMPs being pivotal in this process^3^. In healthy physiology, the ECM environment is strictly regulated through a balance between MMPs and their endogenous inhibitors, known as tissue inhibitors of metalloproteinases (TIMPs)^5^. Specifically, TIMP-1 is vital for binding with one-to-one stoichiometry to almost all MMPs, thus inhibiting their proteolytic functions. In carcinogenesis, the disruption of the MMP/TIMP balance leads to altered ECM composition, which can promote tumor development and increase the likelihood of distant metastasis^4,6,7^. Therefore, the MMP/TIMP balance may more specifically reflect the ECM environment in malignant lesions^6^.

The involvement of MMPs in cancers has been the subject of extensive research. MMPs are often overexpressed in various cancer sites^8–10^. Their overexpression at the tumor site is often associated with poor prognosis^3–5,7,11^. Elevated tissue levels of MMPs may lead to the release of these molecules into the bloodstream, where they can have role as indicators for various diseases^12^. Specifically, concentrations of MMP-8 have been reported to be higher in serum samples from patients with breast, colorectal, and liver cancers^6,13^.

Besides inhibiting the catalytic activities of MMPs, TIMPs have complex, both MMP-dependent and -independent, actions during tumor progression and angiogenesis^14^. TIMP-1 regulates apoptosis and is able serve as a growth factor thus promoting carcinogenesis^15,16^. Elevated levels of TIMPs, particularly TIMP-1, have also been associated with poor prognosis and response to treatment in breast and colorectal cancers^15,17^.

Despite promising findings regarding the potential of MMP-8 and TIMP-1 as diagnostic or prognostic markers in oncology, further research is necessary to validate their potential in clinical applications. Our study aims to examine the association of circulating concentrations of MMP-8 and TIMP-1, as well as the MMP-8/TIMP-1 ratio, with prevalent or incident cancer in a large population-based cohort with a follow-up of 26 years.

## Materials and methods

### Study population

This study is based on the FINRISK97 study, a national population-based cohort (n = 8448) with participants 25 to 74 years of age at baseline^18^. Participants were followed up via registers for 26 years. The study was approved by the Ethics Committee of the National Public Health Institute (number 38/96) and carried out according to the Declaration of Helsinki. All participants have signed an informed consent.

### Cancer data

Participants were followed using record linkage with the Finnish Cancer Registry for cancer registrations. Individuals were defined as cancer cases according to either the type of cancer first registered during their lifetime or as reported in their death certificate. Cancers registered in the study population since the start of the cancer registry in Finland in 1953 were included in the analyses^19^. Highly frequent non-melanoma skin cancer cases were regarded as cancer-free due to the benign characteristics of the disease (n = 200). Cancer events were encoded using ICD-O-3 (International Classification of Diseases) and the cancer outcomes were categorized into any incident cancer (n = 1618) and breast (C50; n = 225), colorectal (C18-20; n = 172), lung (C34; n = 164), and prostate (C61; n = 347) cancers. Information related to cause of death was retrieved from cause of death register provided by Statistics Finland. Death by cancer was considered, if either immediate, underlying or contributing cause of death was cancer.

### Baseline data collection

The subjects filled in a questionnaire and participated in a clinical examination. Body mass index (BMI) was measured in the clinical examination, and smoking habits were recorded using the questionnaires^18^.

### Blood collection and sample preparation

Blood sample collection was made in public health centers and other survey sites. In the invitation letter, the survey participants were asked to fast for at least 4 h and avoid heavy meals before giving the blood sample.

### Serum determinations

The baseline serum concentrations of MMP-8 and TIMP-1 were measured according to manufacturer’s instructions by following assays: active MMP-8 (time-resolved IFMA, Medix Biochemica, Kauniainen, Finland) and TIMP-1 (Chemiluminescent micro particle immunoassay (CMIA), Abbott, Architect i2000). The inter-assay coefficients of variation (CV%) of MMP-8 and TIMP-1 assays were 7.3 and 3.1% respectively.

### Statistical analysis

Statistical analyses were performed with R version 4.2.2 (r-project.org). Before the analysis, MMP-8 and TIMP-1 values were log10-transformed due to the skewed distributions. The significance of differences in baseline characteristics between the individuals without and with prevalent and incident cancer were tested with t-test for continuous and Chi-square test for categorical variables. Differences in mean MMP-8 and TIMP-1 concentrations between cancer groups were assessed with Mann Whitney test. Individuals with prevalent cancer (n = 186) were excluded from the analyses of incident cancer. The associations of MMP-8, TIMP-1, and MMP-8/TIMP-1 concentrations with incident cancer endpoints were analyzed using Cox proportional hazards models adjusted for age, sex, smoking (no, ex or current), and BMI (normal weight, overweight or obese). Proportionality of hazards was tested using Schoenfeld residuals. Age was a violating factor in the models (Supplementary tables) and therefore patients were stratified into five evenly spaced age groups: 25–34, 35–44, 45–54, 55–64 and 65–74 years. In all analyses, a *p*-value below 0.05 was considered statistically significant. Continuous survival plots (package ‘contsurvplot’ in R) were used to visualize probability for cancer incidence and mortality^20^.

## Results

Characteristics of participants with or without incident cancer are presented in Table 1. In total, 1618 (24%) participants who were cancer-free (n = 6644) at baseline were diagnosed with incident cancer during the follow-up. At baseline, patients with incident cancer were older (55.4 vs. 47.1 years; *p* < 0.001) and predominantly male (58% vs 49%; *p* < 0.001) in comparison to cancer-free participants. In addition, the patients with incident cancer had higher BMI than cancer-free participants (*p* < 0.001 for all). There were more smokers and ex-smokers among the cancer patients (*p* < 0.001 for both).

**Table 1:** Characteristics of participants with and without incident cancer.

	With (n = 1618)	Without (n = 6644)	*p*-value*
	Mean (SD)		
Age at baseline (years)	55.4 (11.2)	47.1 (13.5)	**1.2E-132**
	n (%)		
Sex (males)	942 (58.2)	3225 (48.5)	**4.1E-12**
Sex** (males)	601 (56.9)	3225 (48.5)	**5.3E-7**
Smoking			**2.9E-6**
No-smoker	795 (49.1)	3714 (55.9)	
Ex-smoker	416 (25.7)	1417 (21.3)	
Current smoker	407 (25.2)	1509 (22.7)	
BMI			**1.1E-16**
Normal weight	474 (29.3)	2680 (40.3)	
Overweight	390 (44.4)	1206 (39.4)	
Obese	390 (24.1)	1206 (18.2)	

*T-test for continuous variables, Chi-squared test for categorical variables.

**Breast and prostate cancer patients excluded.

Bold indicates statistical significance (*p* < 0.05).

Individuals with prevalent cancer at baseline were excluded.

BMI, body mass index.

Patients with prevalent cancer at baseline had higher serum TIMP-1 levels (*p* < 0.001) than cancer-free participants (Table 2). However, serum MMP-8 levels and MMP-8/TIMP-1 ratios did not differ between these groups.

**Table 2:** MMP-8 and TIMP-1 serum concentrations and MMP-8/TIMP-1 ratio in participants with and without prevalent cancer.

	With (n = 186)	Without (n = 8262)	*p*-value*
	Mean (SD)		
MMP-8	52.1 (69.6)	50.0 (66.4)	0.89
TIMP-1	97.2 (21.5)	90.4 (23.6)	**0.00068**
MMP-8/TIMP-1	0.53 (0.76)	0.59 (0.82)	0.072

Bold indicates statistical significance (*p* < 0.05).

*Mann Whitney test.

Baseline TIMP-1 levels were significantly higher in participants with any incident cancer (*p* < 0.001) (Table 3). In addition, MMP-8/TIMP-1 ratio was lower in any incident cancer patients (*p* = 0.014). Subgroups of patients with incident colorectal and lung cancer had higher TIMP-1 levels (*p* = 0.002 and *p* < 0.001 respectively) compared to participants who remained cancer-free during the follow-up. However, with breast and prostate cancer no significant difference was found. MMP-8 levels were lower in colorectal and prostate cancer patients (*p* = 0.047 and *p* = 0.04), whereas there were no significant differences in subgroups for MMP-8/TIMP-1 ratio.

**Table 3:** Serum concentrations of MMP-8, TIMP-1, and MMP-8/TIMP-1 ratios in participants with or without any incident cancer or breast, colorectal, lung or prostate cancer.

	MMP-8	TIMP-1	MMP-8/TIMP-1 ratio
	Mean (SD), ng/ml		
*Any incident cancer (n = 1618)*			
Yes	47.7 (58.8)	95.4 (26.4)	0.54 (0.67)
No	50.5 (68.1)	89.1 (22.7)	0.60 (0.85)
*p*-value	0.51	**6.7E-26**	**0.014**
*Breast cancer (n = 225)*			
Yes	52.3 (69.1)	88.2 (19.0)	0.61 (0.70)
No	52.5 (59.8)	87.0 (19.8)	0.64 (0.88)
*p*-value	0.34	0.15	0.44
*Colorectal cancer (n = 172)*			
Yes	42.9 (37.8)	94.4 (21.6)	0.48 (0.43)
No	50.1 (66.8)	90.3 (23.7)	0.59 (0.83)
*p*-value	**0.047**	**0.002**	0.064
*Lung cancer (n = 164)*			
Yes	52.4 (68.9)	98.2 (23.5)	0.58 (0.84)
No	49.9 (66.3)	90.20 (23.6)	0.59 (0.82)
*p*-value	0.061	**2.7E-7**	0.24
*Prostate cancer (n = 347)*			
Yes	43.6 (60.6)	92.9 (19.9)	0.50 (0.67)
No	47.8 (64.3)	93.7 (27.0)	0.54 (0.78)
*p*-value	**0.04**	0.31	0.11

Bold indicates statistical significance (*p* < 0.05).

Individuals with prevalent cancer at baseline were excluded.

Mann Whitney test used for statistical analysis.

Breast cancer patients include only females and prostate cancer only males.

During 26-year follow-up in the age-stratified analyses, TIMP-1 levels were significantly associated with any incident cancer in age groups of 55–64 and > 65 years old when adjusted for sex, BMI and smoking (HR 95% CI 6.36, 2.50–16.16 and 6.23, 1.67–23.21 respectively; Table 4). MMP-8 and MMP-8/TIMP-1 ratio associated significantly with cancer in only one age group of 45–54 years old (HR 95% CI 1.37, 1.10–1.71 and 1.31, 1.05–1.64 respectively). When association with cancer mortality was examined in similar age-stratified model, there was a statistically significant association in age groups of 55–64 and > 65 years old for TIMP-1 (HR 95% CI 29.69, 8.25–106.89 and 18.44, 3.64–93.56; Table 5). In age group of 45–54 years old, MMP-8 and MMP-8/TIMP-1 ratio had significant associations with cancer mortality (HR 95% CI 1.65, 1.12–2.44 and 1.54, 1.04–2.27). In younger age groups (< 44 years old) there were no significant associations with cancer incidence or mortality for MMP-8, TIMP-1 and their ratio.

**Table 4:** Association of serum MMP-8, TIMP-1 serum concentrations and MMP-8/TIMP-1 ratios with cancer incidence in age subgroups during 26-year follow-up.

Age group (years)	n	MMP-8	TIMP-1	MMP-8/TIMP-1 ratio
		HR (95% CI)		
25–34	91	1.00 (0.62–1.58)	6.61 (0.84–52.05)	0.94 (0.59–1.51)
35–44	203	1.02 (0.73–1.44)	2.64 (0.56–12.39)	0.97 (0.70–1.34)
45–54	385	**1.37 (1.10–1.71)**	2.68 (0.88–8.14)	**1.31 (1.05–1.64)**
55–64	532	1.21 (0.99–1.48)	**6.36 (2.50–16.16)**	1.13 (0.93–1.39)
65–74	937	1.00 (0.76–1.32)	**6.23 (1.67–23.21)**	0.92 (0.70–1.22)

Bold indicates statistical significance (*p* < 0.05). Cox regression analyses were adjusted for sex, smoking (no, ex or current), and BMI (normal weight, overweight or obese).

Individuals with prevalent cancer at baseline were excluded.

HRs for log10-transformed concentrations.

n = number of incident cancers in age groups.

**Table 5:** Association of MMP-8, TIMP-1 serum concentrations and MMP-8/TIMP-1 ratios with cancer death in age subgroups during 26-year follow-up.

Age group at baseline (years)	n	MMP-8	TIMP-1	MMP-8/TIMP-1 ratio
		HR (95% CI)		
25–34	16	2.14 (0.71–6.47)	27.9 (0.34–2262)	1.75 (0.58–5.28)
35–44	42	1.02 (0.49–2.14)	2.80 (0.10–79.24)	0.99 (0.48–2.04)
45–54	124	**1.65 (1.12–2.44)**	2.60 (0.38–18.02)	**1.54 (1.04–2.27)**
55–64	253	1.32 (0.99–1.77)	**29.69 (8.25–106.89)**	1.11 (0.83–1.48)
65–74	184	1.04 (0.72–1.84)	**18.44 (3.64–93.56)**	0.93 (0.65–1.34)

Bold indicates statistical significance (*p* < 0.05). Cox regression analyses were adjusted for sex, smoking (no, ex or current), and BMI (normal weight, overweight or obese).

Individuals with prevalent cancer at baseline were excluded.

HRs for log10-transformed concentrations.

n = number of cancer deaths in age groups.

**Fig. 1:**
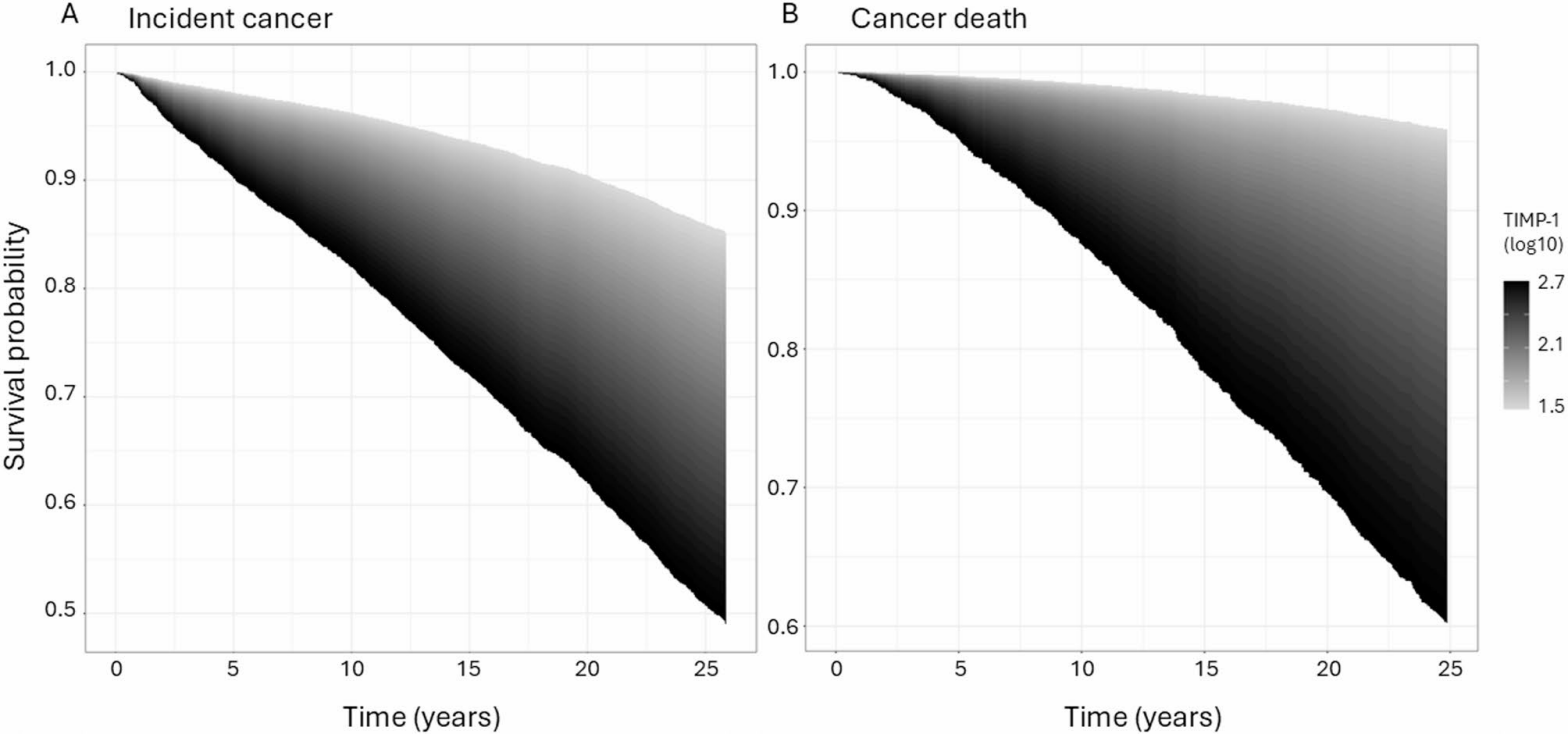
Continuous survival plots for TIMP-1 (log10 concentration) and (**A**) Incident cancer and (**B**) Cancer death. Cumulative probability for patients (y-axis, 0–100%) against time (x-axis, years) is shown. Patients with prevalent cancer were excluded. Models were adjusted for age, sex, smoking (no, ex, current), and BMI (normal weight, overweight, obese).

Survival probability for cancer incidence and mortality over time in relation to TIMP-1 concentration is visualized in Fig. 1 A and B, respectively.

## Discussion

In this large Finnish prospective cohort of 8448 participants followed for 26 years, elevated serum TIMP-1 levels were independently associated with both incident cancer and cancer-related mortality, supporting TIMP-1 as a clinically relevant biomarker of long-term cancer risk. Serum MMP-8 was also associated with cancer incidence, although with a substantially weaker effect size. Adjustment for established risk factors confirmed the robustness of these associations^21,22^. While underlying mechanisms were not examined in this study, previous experimental and clinical evidence suggests that TIMP-1 may influence carcinogenesis through effects on extracellular matrix remodelling, cell survival, and angiogenesis^21–23^, providing biological plausibility for the observed associations.

TIMP-1 serum concentrations were higher in both prevalent and incident cancer patients. Furthermore, significantly higher serum concentrations were observed in colorectal and lung cancer. Our result support the previous findings of elevated serum and plasma TIMP-1 levels in multiple cancer types, including colorectal and lung cancer^6,17,21,22,24–26^. However, in our data TIMP-1 concentrations were not significantly different in prostate and breast cancer. The absence of statistical significance may reflect sex hormone–dependent regulation of the TIMP-1 gene, with higher hormone levels associated with elevated baseline TIMP-1 concentrations^27,28^. However, this could not be assessed in the present study.

TIMP-1 was associated significantly with cancer incidence and cancer-related mortality in age-stratified Cox regression model. This association was observed in older age groups (55–64 and > 65 years), which is consistent with the well-documented exponential increase in cancer incidence after the age of 50^1^. Although TIMPs are classically described as inhibitors of pro-tumorigenic and pro-metastatic MMPs and might therefore be expected to exert protective effects, accumulated evidence indicates that their biological functions are multifaceted and, in many contexts, more tumor-promoting than protective. Previous reports have demonstrated that TIMP-1 is especially associated with metastasis and cancer-related cachexia, features commonly observed in patients with poor survival^24,29–31^.

In our study, serum concentration of MMP-8 was significantly lower in participants with colorectal and prostate cancer, but no significant changes were found in any other cancer types. These results conflict with previous studies showing increased serum levels of MMP-8 patients with breast, colorectal and liver cancers^6,13,32^. Despite some significant associations observed in any incident cancer and in breast and prostate cancers, there were no associations between the MMP-8/TIMP-1 ratio and either prevalent or incident cancers or with cancer mortality. The weak associations of MMP-8 and MMP-8/TIMP-1 ratio with cancer incidence and mortality can be attributed to the diverse roles of MMPs in carcinogenesis, encompassing both tumor-promoting and tumor-suppressing actions^13,33^. This might dilute the results in our large data set. In addition, it has been previously shown that MMP-8 concentrations decrease with age, which may influence their association with cancer especially in studies that have not been stratified for age^22^.

Chronic inflammation is involved in multiple pathologies, including cancers^34^. Indeed, epidemiological data suggests that over a quarter of all cancers are associated with chronic inflammation^35^. Increased serum concentrations of MMP-8 have been frequently reported in inflammatory conditions such as periodontitis, rheumatoid arthritis, and infectious diseases^13,36,37^. Thus, it has been suggested that the inflammatory promotion in cancer development may be mediated by increased MMP activity^35^. In addition, TIMP-1 may contribute to inflammatory responses by activating monocytes and neutrophils and promoting cytokine production^32^. This mechanism could partially explain the elevated TIMP-1 levels and the associated increased cancer risk and poor prognosis reported by this study and other researchers^21–23^. Obesity and smoking should also be considered in this context, as both are well-established proinflammatory factors^38,39^. These variables were therefore accounted for in our analyses to minimize potential confounding.

The use of large national and well-characterized population-based cohort in combination with well-documented national cancer registry data can be considered as a strength of this study. This is the first study to assess MMP-8 and TIMP-1 serum concentrations in cancer patients in such a large population and time span. However, it is essential to acknowledge its limitations as well. Pre-analytical issues concerning the use of blood, especially serum, in tumor marker studies have been discussed^40,41^. The use of serum samples may lead to release of MMPs from platelets and leukocytes during the ex vivo clotting process, resulting higher levels of MMPs in serum samples compared to corresponding plasma samples^40,42,43^. Similarly, it has been suggested that TIMP-1 levels may be elevated due to the release of this enzyme from platelets, which contain TIMP-1 stored in their alpha-granules^44^. However, there is a strong correlation between serum and plasma MMP-8 levels^45,46^. We also acknowledge that not all of the potential confounding factors, such as dietary factors and alcohol use, were assessed as the data was not available across the whole data set.

In conclusion, the data presented here supports and extends existing evidence that MMPs and TIMPs are involved in cancer development and progression likely through mechanisms related to ECM remodeling and immunomodulation. In this large prospective cohort, higher serum TIMP-1 levels were significantly associated with increased cancer incidence and cancer-related mortality. These findings contribute to the growing body of evidence implicating ECM-related pathways in cancer biology, and suggest that especially TIMP-1 may have potential as a biomarker for cancer risk. Further research is required to clarify the underlying mechanisms and to determine the clinical relevance and utility of TIMP-1 and related markers in cancer risk assessment, prevention, and management.

## Supplementary Information

Below is the link to the electronic supplementary material.

Supplementary Material 1.

## Data Availability

The FINRISK data for the present study is available with a written application to the THL Biobank as instructed on the website of the Biobank (https://thl.fi/en/research-and-development/thl-biobank/for-researchers). A separate permission is needed from FINDATA (https://www.findata.fi/en/) for use of the electronic health record data.
