# Supplementary material for: Serum TIMP-1 concentrations are associated with cancer incidence and cancer-related mortality in a population-based cohort
Source: Sci Rep. 2026 May 20;16:23048. doi: 10.1038/s41598-026-52354-5 (PMC13392385; doi:10.1038/s41598-026-52354-5)
Supplement: Supplementary file 1 — Supplementary Material 1. [file 41598_2026_52354_MOESM1_ESM.pdf]

SUPPLEMENTARY INFORMATION

Supplementary tables

Supplementary Table 1. Association of serum MMP-8, TIMP-1 concentrations and MMP-8/TIMP-1 ratios with incidence of breast, colorectal, lung, prostate or any cancer during 26-year follow-up.

		Incident cancer				
		**Any**	**Breast**	**Colorectal**	**Lung**	**Prostate**
**MMP-8**	HR	1.20	1.19	1.22	1.27	1.01
	95% CI	1.06-1.35	0.87-1.63	0.85-1.75	0.87-1.84	0.77-1.31
	p-value	0.003	0.27	0.29	0.22	0.97
**TIMP-1**	HR	4.50	2.50	3.61	6.49	0.27
	95% CI	2.58-7.85	0.52-11.98	0.65-20.19	1.10-38.35	0.07-1.02
	p-value	**2.1E-7**	0.22	0.14	**0.03**	0.06
**MMP-8 /TIMP-1 ratio**	HR	1.12	1.13	1.13	1.17	1.10
	95% CI	0.99-1.26	0.83-1.55	0.79-1.63	0.80-1.71	0.85-1.43
	p-value	0.05	0.44	0.50	0.41	0.48

Bold indicates statistical significance ($p < 0.05$). Cox regression analyses were adjusted for age, sex, smoking (no, ex or current), and BMI (normal weight, overweight or obese). Individuals with prevalent cancer at baseline were excluded. HRs for log10-transformed concentrations. Breast cancer analyzed only in females and prostate cancer only in males.

Supplementary Table 2. Association of serum MMP-8 and TIMP-1 concentrations, and MMP-8/TIMP-1 ratios with cancer death during 26-year follow-up.

		No prevalent cancer	Incident cancer & no prevalent cancer
**MMP-8**	HR	1.31	1.23
	95% CI	1.08-1.57	1.01-1.49
	p-value	**0.005**	**0.04**
**TIMP-1**	HR	12.63	6.64
	95% CI	5.41-29.46	2.91-15.16
	p-value	**4.4E-9**	**5.2E-6**
**MMP-8/TIMP-1 ratio**	HR	1.16	1.11
	95% CI	0.96-1.40	0.92-1.34
	p-value	0.12	0.28

Bold indicates statistical significance ($p < 0.05$). Cox regression analyses were adjusted for age, sex, smoking (no, ex or current), and BMI (normal weight, overweight or obese). HRs for log10-transformed concentrations.
